# Decreasing β-Catenin Leads to Altered Endothelial Morphology, Increased Barrier Permeability and Cognitive Impairment During Chronic Methamphetamine Exposure

**DOI:** 10.3390/ijms26041514

**Published:** 2025-02-11

**Authors:** Hai Qiu, Manting Zhang, Chuanxiang Chen, Huijun Wang, Xia Yue

**Affiliations:** Guangzhou Key Laboratory of Forensic Multi-Omics for Precision Identification, School of Forensic Medicine, Southern Medical University, Guangzhou 510515, China; tzonerqiuhai@i.smu.edu.cn (H.Q.); zmt1996@i.smu.edu.cn (M.Z.); chanel00@smu.edu.cn (C.C.)

**Keywords:** methamphetamine, chronic exposure, cognitive impairment, β-catenin, endothelial cells, blood–brain barrier injury

## Abstract

Cognitive impairment induced by chronic methamphetamine (METH) exposure exhibits similarities to neurodegenerative disorders and is associated with blood–brain barrier (BBB) dysfunction. However, the potential involvement of β-catenin in maintaining BBB integrity during METH exposure remains unexplored. In this study, Y-maze and novel object recognition tests were conducted to assess cognitive impairment in mice exposed chronically to methamphetamine for 2 and 4 weeks. Gd-DTPA and Evans blue leakage tests revealed disruption of the BBB in the hippocampus, while chronic METH exposure for 2 and 4 weeks significantly decreased β-catenin levels along with its transcriptionally regulated protein, claudin5. Additionally, various neural injury-related proteins, such as APP, Aβ1–42, p-tau (Thr181) and p-tau (Ser396), as well as neuroinflammation-related proteins, such as IL-6, IL-1β, and TNF-α, exhibited increased levels following chronic METH exposure. Furthermore, plasma analysis indicated elevated levels of p-Tau (total), neurofilament light chain, and GFAP. In vitro experiments demonstrated that exposure to METH resulted in dose-dependent and time-dependent reductions in cellular activity and connectivity of bEnd.3 and hcmec/D3 cells. Furthermore, β-catenin exhibited decreased levels and altered subcellular localization, transitioning from the cell membrane to the cytoplasm and nucleus upon METH exposure. Overexpression of β-catenin was found to alleviate endothelial toxicity and attenuate junctional weakening induced by METH. The aforementioned findings underscore the crucial involvement of β-catenin in endothelial cells during chronic METH exposure-induced disruption of the BBB, thereby presenting a potential novel target for addressing METH-associated cerebrovascular dysfunction and cognitive impairment.

## 1. Introduction

Methamphetamine (METH) is one of the most prevalent stimulant drugs nationally and globally, causing a series of public health and social problems, including high mortality rate, relapse rate and crime rate, in turn leading to a loss of labor force and increasing social instability [1]. METH abuse gives rise to serious addiction in individuals with substance dependence and leads to poisonous organic damage to important human organs, especially the central nervous system [2]. According to clinical and forensic case reports, acute METH poisoning can arouse hyperthermia, mental disorder, excessive mechanical activity, high blood pressure, cerebral hemorrhage, myocardial ischemia, fatal arrhythmia, etc., while chronic METH poisoning may be related to symptoms of paranoia, depression, delusion, schizophrenia or other psychiatric disorders, and cerebral lesions [3]. Many studies have indicated that some of the lesions caused by METH abuse, especially chronically, have some clinically or pathologically similarities to those found in patients with Alzheimer’s disease (AD) [4,5,6]. Both can be characterized by cognitive impairment, neuronal damage, neuroinflammation, progressive neurovascular dysfunction, disruption of the blood–brain barrier (BBB), metabolic disorders, and the accumulation of harmful substances of pathological proteins like β-amyloid and phosphorylated tau.

BBB injury is an important pathological mechanism of AD and METH neural injury [7,8]. Endothelial cells (ECs) of the BBB have distinct barrier properties to maintain brain homeostasis, controlling the entry of blood components into the brain or the elimination of metabolic waste and toxic molecules. In aging or METH poisoning, dysfunction of ECs has manifested as capillary leakage, which contributes to the accumulation of pathological protein, activation of glial cells, neuroinflammation and neuronal injury or loss, and eventually leads to neurodegenerative disease [8]. Previous studies indicate that METH can injure ECs and lead to structural damage of the BBB [9], abnormal substance transport [10], increased migration of leukocytes [11] and release of their inflammatory cytokine [12], and triggers an inflammatory response in glial cells, releasing inflammatory factors (e.g., TNF-α, IL-6, IL-1β), which indirectly affects neurological function and causes AD-like cognitive impairment. METH may cause EC damage and destroy BBB by various mechanisms, including oxidative stress, apoptosis, necrosis, and increased matrix metalloproteinase 9 (MMP9) release, etc. [7]. However, the mechanism of METH’s toxic effects is extensive and complicated. Current studies focus mostly on the acute or subacute effects of METH, the lack of elucidating chronic effects, and on the tight junction, neglecting the fact that the adherens junction is a prerequisite for the tight junction and is important to the BBB. In addition, whether BBB injury is sustained remains controversial [13,14,15]. β-catenin functions as an essential component of cadherin-mediated cell–cell adhesion, contributing to the establishment of polarized epithelial tissues and the maintenance of BBB integrity. Additionally, the β-catenin-dependent Wnt signaling pathway plays a crucial role in angiogenesis, BBB formation, and functional maintenance. Endothelial β-catenin is pivotal for embryonic and postnatal BBB maturation by regulating tight junction (TJ) assembly. However, the precise function of β-catenin in METH research remains elusive [16,17].

This study aims to provide evidence of cognitive impairment and BBB injury caused by chronic METH exposure, and to explore the effects of METH on endothelial adherens junction protein β-catenin. Through this study, we aim to provide novel toxicological insights and evidence for METH BBB injury, leading to tangible benefits for abusers.

## 2. Results

### 2.1. Chronic METH Exposure Resulted in Reduced Cognitive Function in Mice

To investigate the cognitive function of chronic METH-abused mice, we conducted Y-maze and NOR tests after 2 weeks and 4 weeks of METH exposure.

**Figure 1:**
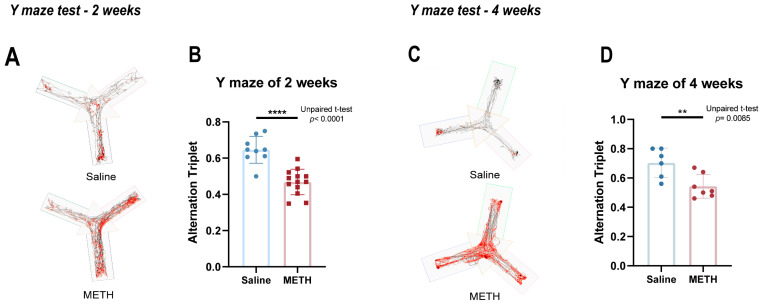
The Y-maze behavioral results of mice after 2 and 4 weeks of exposure to chronic METH. (**A**) Y-maze trace trajectories and heatmap profiling at 2 weeks post-exposure. (**B**) The spontaneous alternation behavior scores of mice in Y-maze at 2 weeks post-exposure. (**C**) Y-maze trace trajectories and heatmap profiling at 4 weeks post-exposure. (**D**) The spontaneous alternation behavior scores of mice in Y-maze at 4 weeks post-exposure. The statistical significance is shown as ** *p* < 0.01, **** *p* < 0.0001.

The results of the Y-maze experiments conducted 2 weeks post-exposure revealed a significant reduction in the spontaneous alternation behavior score of METH-treated mice compared with the saline group (saline: *n* = 6, 0.6500 ± 0.01949; Shapiro–Wilk test, w = 0.8033, *p* = 0.0630; METH: *n* = 11, 0.4767 ± 0.02033; Shapiro–Wilk test, w = 0.96323, *p* = 0.8026; unpaired t-test, *p* = 0.0001) (Figure 1A,B). Similarly, 4 weeks post-exposure, the Y-maze experiment also demonstrated a significant decrease in the spontaneous alternation behavior score in METH-treated mice relative to the saline group (saline: *n* = 6, 0.7033 ± 0.04088; Shapiro–Wilk test, w = 0.897, *p* = 0.3565; METH: *n* = 7, 0.5429 ± 0.03061; Shapiro–Wilk test, w = 0.8697, *p* = 0.1846; unpaired t-test, *p* = 0.0085) (Figure 1C,D).

**Figure 2:**
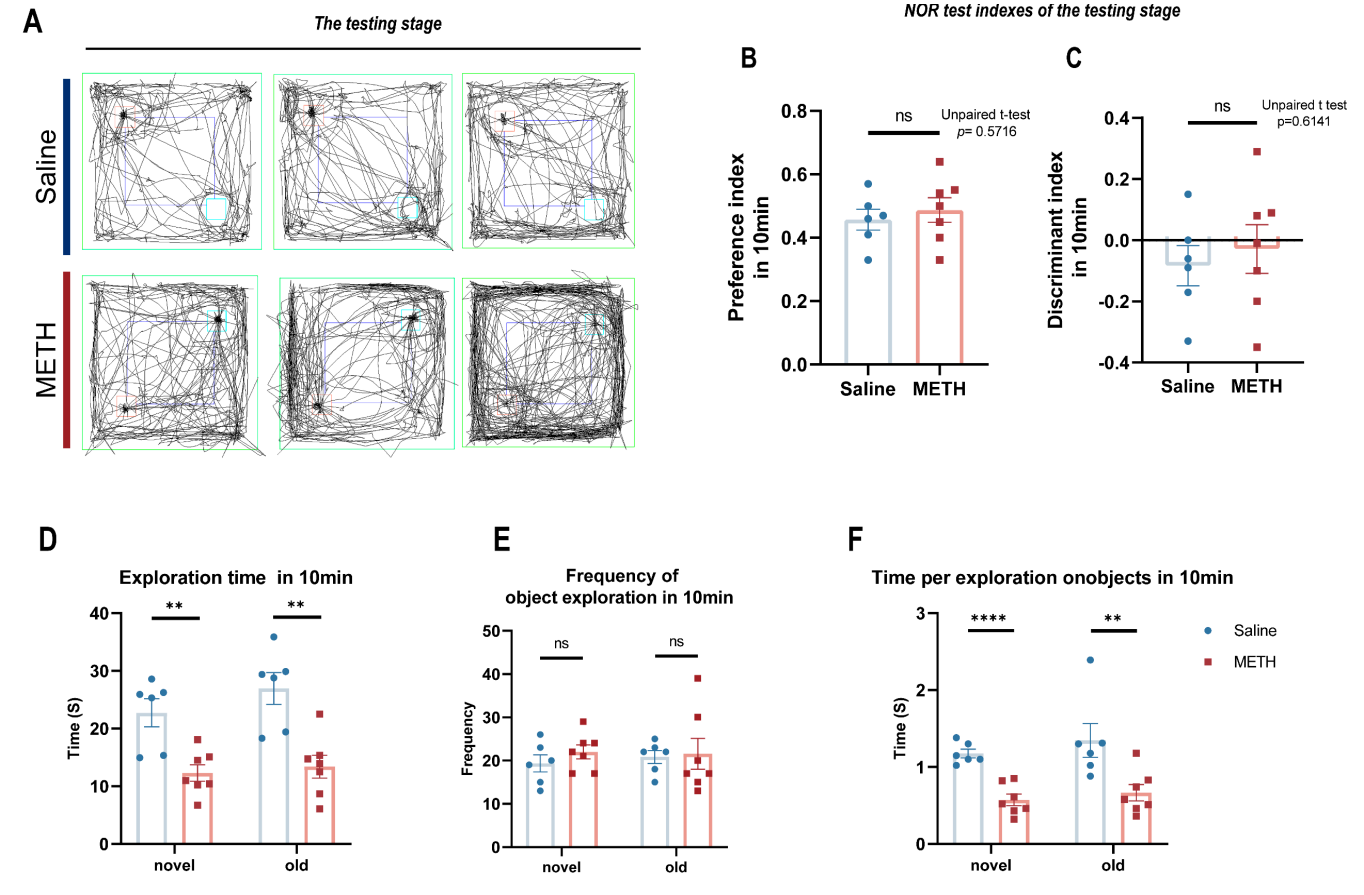
The NOR results of mice after 4 weeks of exposure to chronic METH. (**A**) Representative pictures of mice movement trajectories in the testing stage of the 4-week NOR test (The light blue rectangles represent novel objects, the red ones represent old ones, and the dark blue ones indicate the central area.). (**B**) Reference index of mice in NOR at 4 weeks post-exposure. (**C**) Discrimination index of mice in NOR at 4 weeks post-exposure. (**D**) The total time to explore novel and familiar object. (**E**) The exploration frequence of novel and familiar object. (**F**) The average time spent in exploration on novel and familiar objects. The statistical significance is shown as ns *p* > 0.05, ** *p* < 0.01, **** *p* < 0.0001.

The movement trajectories of mice in the testing stage of the NOR test at 4 weeks post-exposure to METH are shown in Figure 2A. Although no significant differences were observed between the groups in the discrimination index (saline: *n* = 6, 0.4567 ± 0.03323; METH: *n* = 7, 0.4871 ± 0.03902; unpaired t-test *p* = 0.5716) (Figure 2B), or the preference index (saline: *n* = 6, 0.4567 ± 0.03323; METH: *n* = 7, 0.4871 ± 0.03902; unpaired t-test *p* = 0.5716) (Figure 2C), when compared with saline groups the total time of novel object exploration of the METH group mice decreased (saline: 22.73 ± 2.438; METH: 12.29 ± 1.439; Mann–Whitney test *p* = 0.0082), while the total time of familiar object exploration also decreased (saline: 26.95 ± 2.761; METH: 13.40 ± 1.983; unpaired t-test *p* = 0.0019) (Figure 2D). Additionally, no statistically significant difference was observed in the exploration frequency between novel and familiar objects (novel object: saline: 19.33 ± 1.978; METH: 22.00 ± 1.604; unpaired t-test *p* = 0.3123 vs. familiar object: saline: 20.83 ± 1.493; METH: 21.57 ± 3.571; unpaired t-test *p* = 0.8610) (Figure 2E). However, significant differences were observed on the average time spent in exploration for novel and familiar objects (novel object: saline: 1.175 ± 0.05584; METH: 0.5729 ± 0.07546; unpaired t-test *p* < 0.0001vs. familiar object: saline: 1.347 ± 0.2194; METH: 0.6657 ± 0.1051; Mann–Whitney test *p* = 0.0052) (Figure 2F).

**Scheme 1:**
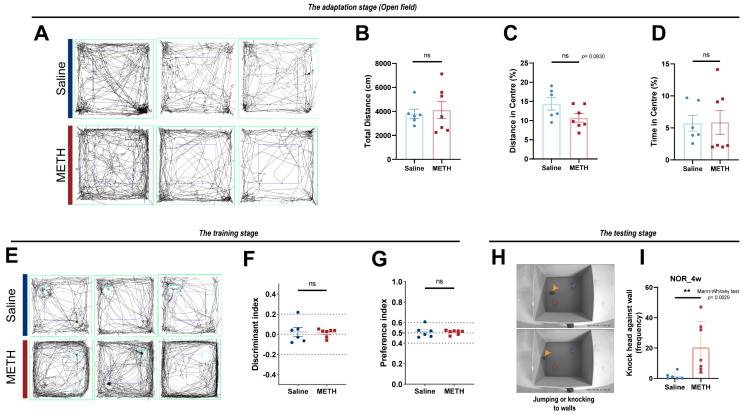
The performance of mice in the NOR test adaptation stage, training stage, and testing stage (The light blue rectangles represent novel objects, the red ones represent old ones, and the dark blue ones indicate the central area.). (**A**–**D**) Representative pictures of mice movement trajectories in the adaptation stage (open field) of the 4-week NOR test, and their statistical analysis of total distance, moving distance in central area (%), and moving time in central area (%). (**E**–**G**) Representative pictures of mice movement trajectories in the training stage, and their statistical analysis of discrimination index and preference index. (**H**,**I**) Some mice exhibited the behavior of knocking against the walls in the testing stage of the 4-week NOR test and relevant statistics (The yellow arrow points to the location of the mouse.). The statistical significance is shown as ns *p* > 0.05, ** *p* < 0.01.

During the adaptation stage, no significant differences were observed in the total distance traveled (saline: 3799 ± 389.0; METH: 4127 ± 712.0; Mann–Whitney test *p* > 0.9999), distance moved in the central area (%) (saline: 14.36 ± 1.604; METH: 10.73 ± 1.111; unpaired t-test *p =* 0.0830), and time spent moving in the central area (%) (saline: 5.730 ± 1.235; METH: 5.886 ± 1.871; Mann–Whitney test *p =* 0.4452) (Scheme 1A–D). Additionally, during the training stage of the NOR test, the exploration discrimination index (saline: 0.02283 ± 0.04504; METH: 0.01529 ± 0.01566; Mann–Whitney test *p =* 0.9452) and preference index (saline: 0.5110 ± 0.02253; METH: 0.5076 ± 0.007852; Mann–Whitney test *p =* 0.9182) of the two groups of mice did not show significant differences (Scheme 1E–G). However, we recorded a significant increase in wall-knocking behavior among METH-treated mice during the testing stage (saline: 1.500 ± 0.9574; METH: 20.43 ± 6.414; Mann–Whitney test *p* = 0.0029) (Scheme 1H,I).

### 2.2. Chronic METH Exposure Induced Neural Injury and Neuroinflammation in the Hippocampus

**Figure 3:**
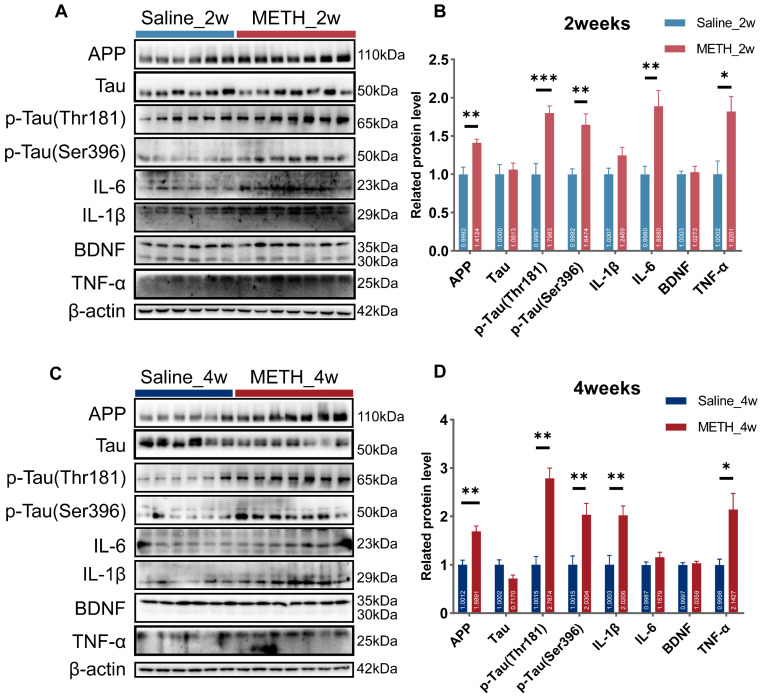
Western blot analysis of neural injury-related proteins in hippocampal tissue. (**A**,**B**) Protein expression profiles in mice exposed to chronic methamphetamine (METH) for 2 weeks. (**C**,**D**) Protein expression profiles in mice exposed to chronic METH for 4 weeks. Statistical comparisons were performed between saline-treated and METH-treated groups at each time point: Saline_2w (*n* = 6) vs. METH_2w (*n* = 7) and Saline_4w (*n* = 6) vs. METH_4w (*n* = 7). Data are expressed as mean ± SEM. Statistical significance was assessed using unpaired two-tailed Student’ s t-tests or Mann–Whitney U tests (for non-normally distributed data). The statistical significance is shown as * *p* < 0.05, ** *p* < 0.01, *** *p* < 0.001.

To investigate hippocampal neural injury, we employed western blot to detect hippocampal neural injury-associated proteins like amyloid precursor protein (APP), microtubule-associated protein tau and its phosphorylated forms (p-tau (Thr181) and p-tau (Ser396)). Additionally, we assessed the expression levels of neuroinflammatory factors like IL-6, IL-1β and TNF-α, and brain-derived neurotrophic factor (BDNF). In the hippocampi of mice exposed to METH for 2 weeks, there was a significant increase in the protein expression levels of APP, p-tau (Thr181), p-tau (Ser396), IL-6, and TNF-α (Figure 3A,B). Similarly, in the hippocampi of mice exposed to METH for 4 weeks, there was also a significant increase in the protein expression levels of APP, p-tau (Thr181), p-tau (Ser396), IL-1β, and TNF-α (Figure 3C,D).

**Figure 4:**
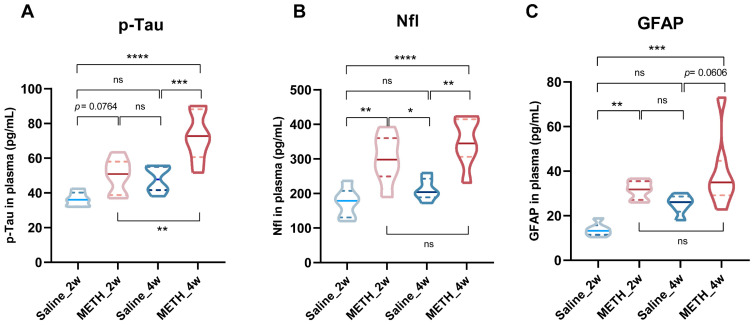
Elisa for p-Tau, NEFL(Nfl), and GFAP in serum of mice administered METH for 2 or 4 weeks. (**A**) Elisa for p-Tau in serum of mice post-administered METH. (**B**) Elisa for Nfl in serum of mice post-administered METH. (**C**) Elisa for GFAP in serum of mice post-administered METH. Data are expressed as mean ± SEM. Statistical significance was assessed using one-way ANOVA and Tukey HSD test (for multiple comparison test). The statistical significance is shown as ns *p* > 0.05, * *p* < 0.05, ** *p* < 0.01, *** *p* < 0.001, **** *p* < 0.0001.

Given that peripheral blood marker proteins can reflect nerve injury and neuroinflammation in Alzheimer’s disease (AD) and other neurodegenerative diseases, we also examined these plasma markers to assess similar effects in chronic METH-exposed mice. Compared with control groups of the same treatment time (2w or 4w), the p-tau (total), neurofilament light (Nfl/NEFL) and glial fibrillary acidic protein (GFAP) were significantly increased in the plasma of METH group mice (Figure 4A–C).

**Figure 5:**
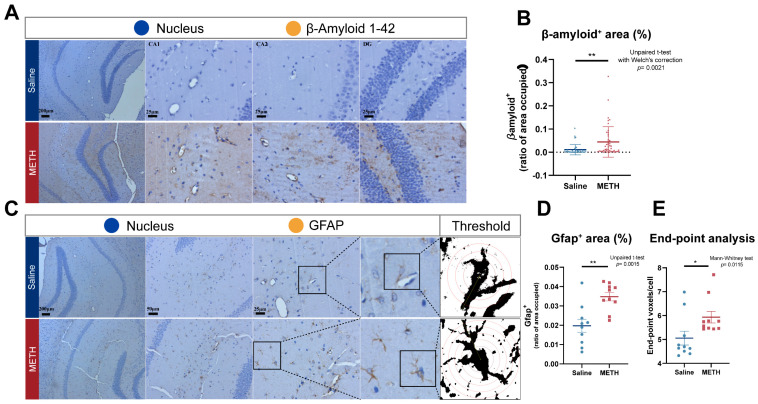
IHC for β-amyloid 1-42 and GFAP in hippocampus of mice administered METH for 2 or 4 weeks. (**A**,**B**) IHC results and statistics of β-amyloid 1-42 of 2-week-treatment mice. Scale bars (from left to right): 200, 25, 25, 25 μm and microscope’s magnification (from left to right): 100×, 400×, 400×, 400×. (**C**–**E**) IHC results and statistics of GFAP of 2-week-treatment mice. Scale bars (from left to right): 200, 50, 25 μm and microscope’s magnification (from left to right): 100×, 200×, 400×. The image of the selected region has been magnified by a factor of two. Picture of threshold shows Sholl analysis of astrocytes to indicate the complexity of cell structure. GFAP^+^ occupied area and the end-point voxels were used for statistics. The statistical significance is shown as * *p* < 0.05, ** *p* < 0.01.

Furthermore, IHC results show that, compared with the control groups, the positive signal area of Aβ1-42 in the METH group were elevated, particularly around blood vessels (Figure 5A,B). The positive signal area of GFAP also exhibited an increase within the hippocampus (Figure 5C) and were characterized by enlarged somata (Figure 5D) along with elongated protrusions (Figure 5E).

### 2.3. Chronic METH Exposure Leads to BBB Leakage and β-Catenin Decreasing in Hippocampus

**Figure 6:**
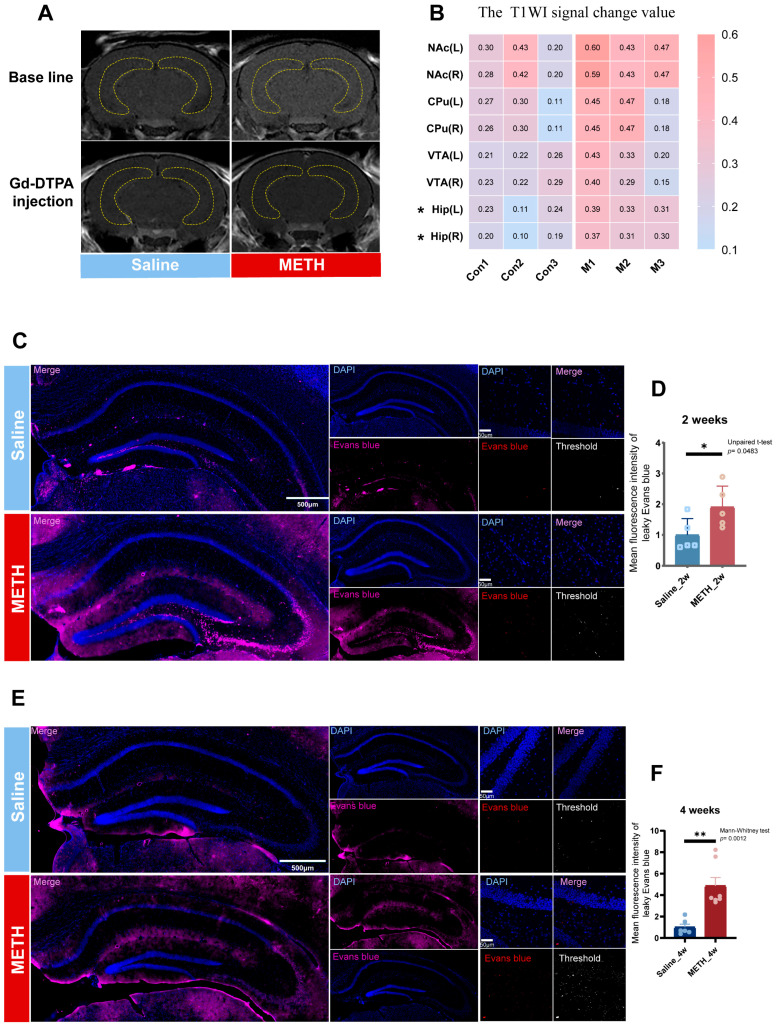
Chronic METH exposure leads to BBB leakage. (**A**,**B**) MRI detection of contrast agent Gd-DTPA leakage in 4 w treatment mice (*n* = 3) (The yellow area represents the representative hippocampal brain regions.) and related statistics (L means left brain, R means right brain; NAc-nucleus accumbens; CPu-corpus striatum; Hip-hippocampus; VTA-ventral tegmental area; *n*_saline_ = 3, *n*_METH_ = 3), the statistical significance is shown as * *p* < 0.05 beside the brain regions. (**C**,**D**) Evans blue leakage or remaining fluorescence detection results and fluorescence intensity quantitative statistics of mice with 2 weeks of METH exposure. The saline group of the same period was set to 1 for standardization and comparison (2 weeks: *n*_saline_ = 5, *n*_METH_ = 5). (**E**,**F**) Evans blue leakage or remaining fluorescence detection results and fluorescence intensity quantitative statistics of mice with 4 weeks of METH exposure. The saline group of the same period was set to 1 for standardization and comparison (4 weeks: *n*_saline_ = 6, *n*_METH_ = 7). For C and E, the complete hippocampal image was generated by stitching together scanned images acquired at 200× magnification, while the partial images were captured at 400× magnification. The statistical significance is shown as * *p* < 0.05, ** *p* < 0.01.

**Scheme 2:**
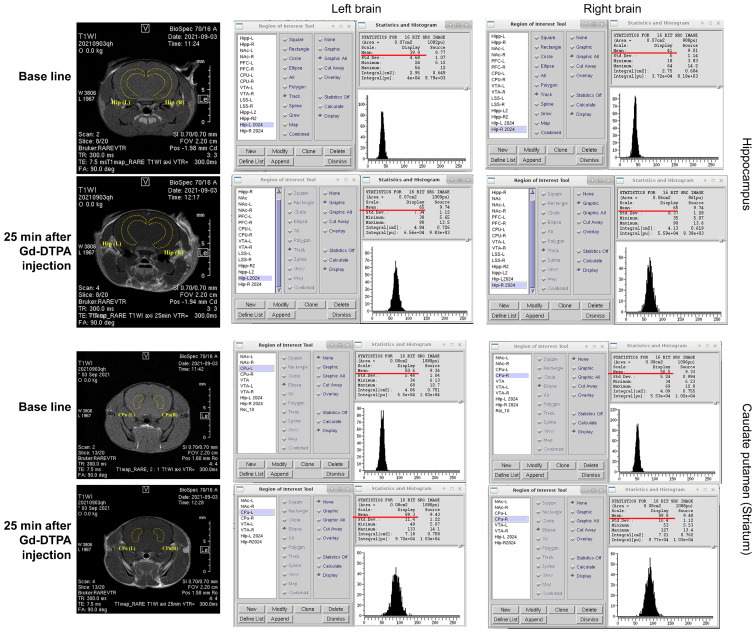
A schematic diagram of mouse MRI-T1-WI imaging and data acquisition. The formula for calculating the signal intensity of the corresponding brain region in T1-WI imaging is as follows: (Mean_25min after injection_ − Mean_Basline_)/Mean_Basline_. (The outlines of the brain region are depicted as the yellow dotted line in the left figures. The mean value is marked with a red underline.).

In this study, we investigated the BBB permeability in the hippocampi of mice subjected to different treatment durations. By employing MRI, we assessed the permeability of the BBB to Gd-DTPA contrast agent in live mice treated for 4 weeks, revealing an increased mean value of T1-WI after injection of GD-DTPA within brain regions, including the hippocampus, nucleus accumbens, striatum, and ventral tegmental area (Figure 6A,B). The process of obtaining and calculating the signal intensity values of the relevant brain regions in T1-WI is shown in Scheme 2. The increase in signal intensity of the hippocampal region on T1-WI is statistically significant (L: difference: −0.1488 ± 0.04943, unpaired t-test *p* = 0.0395; R: difference: −0.1631 ± 0.03958, unpaired t-test *p* = 0.0146). Additionally, we evaluated hippocampal BBB leakage using fluorescence detection of Evans blue dye. Our findings demonstrate that METH chronic exposure for 2 weeks (difference: 0.9036 ± 0.3881; unpaired t-test *p* = 0.0483) (Figure 6C,D) and 4 weeks (difference actual: 3.054; Mann–Whitney test *p* = 0.0012) (Figure 6E,F) resulted in elevated Evans blue leakage from the mouse hippocampus. Notably, there was a substantial increase in fluorescence signal at 4 weeks following METH treatment.

**Figure 7:**
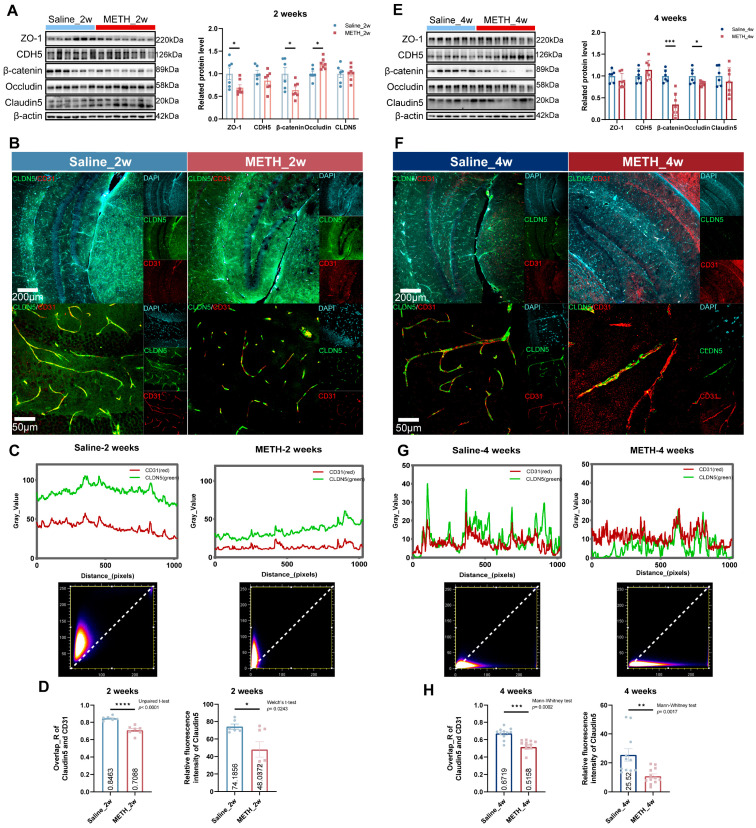
Chronic METH exposure leads to claudin5 and β-catenin reduction, and other cell junction proteins changing. (**A**) Western blot analyses of cell-junction-associated protein in the hippocampi of mice treated for 2 weeks (*n*_saline_ = 6, *n*_METH_ = 7). (**B**) Immunofluorescence staining of claudin5 and CD31 in the hippocampi of mice exposed to METH for 2 weeks. (**C**) Immunofluorescence co-localization analysis of claudin5 and CD31 of mice exposed to METH for 2 weeks. (**D**) Statistics of colocalization coefficient R (*n*_saline_ = 6, *n*_METH_ = 7) and immunofluorescence intensity (*n*_saline_ = 6, *n*_METH_ = 7) of claudin5 for mice exposed to METH for 2 weeks. (**E**) Western blot analyses of cell-junction-associated protein in the hippocampi of mice treated for 4 weeks (*n*_saline_ = 6, *n*_METH_ = 7). (**F**) Immunofluorescence staining of claudin5 and CD31 in the hippocampi of mice exposed to METH for 4 weeks. (**G**) Immunofluorescence co-localization analysis of claudin5 and CD31 of mice exposed to METH for 4 weeks. (**H**) Statistics of colocalization coefficient R (*n*_saline_ = 11, *n*_METH_ = 12) and immunofluorescence intensity (*n*_saline_ = 11, *n*_METH_ = 12) of claudin5 for mice exposed to METH for 4 weeks. Scale bars in each group: 200 μm for upper, and 50 μm for lower. The statistical significance is shown as * *p* < 0.05, ** *p* < 0.01, *** *p* < 0.001, **** *p* < 0.0001.

Subsequently, Western blot analysis was employed to assess the expression of hippocampal connection-related proteins in mice. In comparison with the 2-week saline control group, exposure to METH for 2 weeks led to a significant decrease in ZO-1 and β-catenin expression, as well as a notable increase in occludin expression (Figure 7A). Similarly, when compared with the 4-week saline control group, METH exposure resulted in a significant reduction of β-catenin and occludin expression in the hippocampus of mice exposed to METH for 4 weeks (Figure 7E). Additionally, double immunofluorescence staining was utilized to examine both co-localization and expression levels of claudin5 and Cd31 (a vascular endothelial cell marker) within the hippocampus of 2-week (Figure 7B) and 4-week (Figure 7F) treated mice. By analyzing the longest blood vessel segments tracked in the xyz composite image using ImageJ, we measured the fluorescence signal intensity and signal colocalization level of claudin5 and Cd31 on this trajectory (colocalization level: the higher the pixel signal is along the diagonal, the more colocalization there is) (Figure 7C,G). Our findings revealed that exposure to METH for both 2 weeks (Figure 7D) and 4 weeks (Figure 7H) significantly decreased claudin5 fluorescence colocalization with Cd31 as well as its fluorescence intensity when compared with the corresponding periods of saline control group.

### 2.4. The Increase of METH Concentration Leads to Endothelial Injury and Decreased β-Catenin

**Figure 8:**
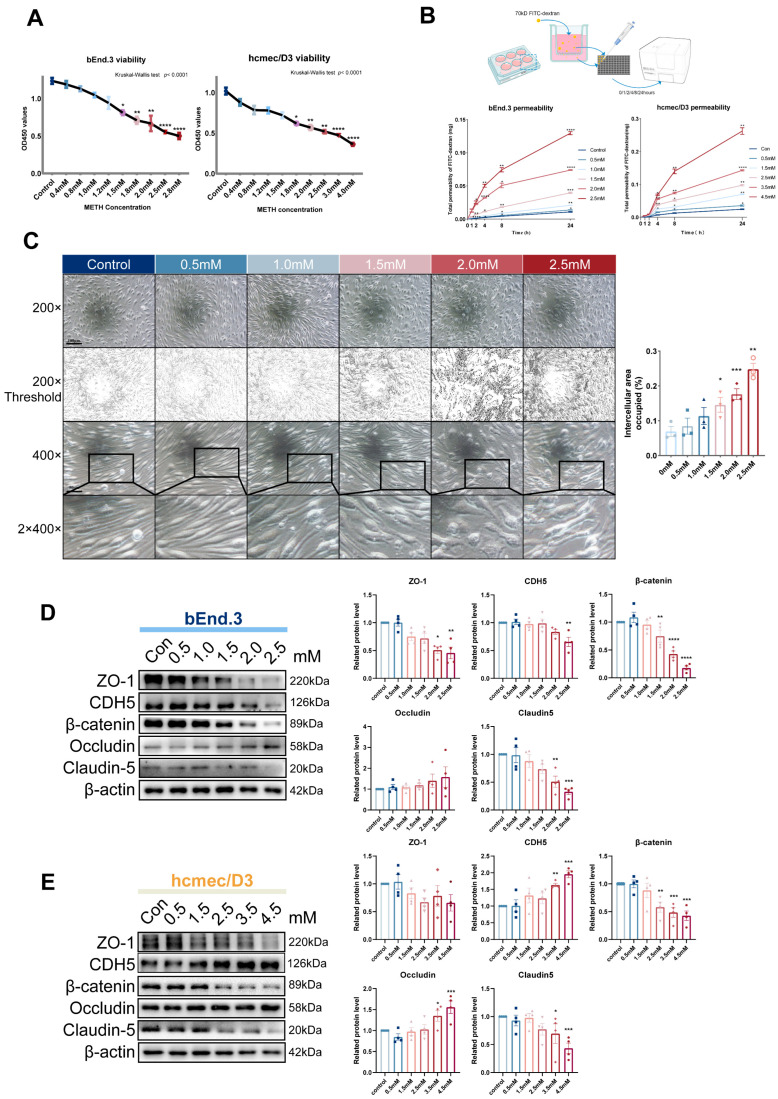
The increase of METH concentration cause endothelial cell injury, permeability increase, decreased β-catenin and changes in other cell junction proteins. (**A**) bEnd.3 (left) and hcmec/D3 (right) cell viability was measured by CCK8 after 24 h treatment with different METH concentration gradients. Kruskal–Wallis test was used to compare between different concentrations of METH vs. control. (**B**) The modeling and detection of 70 kDa FITC-dextran permeability test for monolayer endothelial barrier in the small chamber of a transwell (above). In a bEnd.3 monolayer endothelial barrier (lower left), the total permeation amount of FITC changed under different concentrations of METH (24 h treatment) and different detection time. The same statistics for hcmec/D3 are on the lower right. Two-way ANOVA was used to compare the effect of METH treatment and testing time. (**C**) Cell morphology observation of bEnd.3 treated with METH concentration gradients for 24 h. The second row of images shows the threshold images of the intercellular area at a magnification of 200×, which were used for statistics. Scale bars in 200× and 400× images are shown as 100 and 50 μm. (**D**,**E**) Western blot analyzed cell-junction-related proteins in bEnd.3 and hcmec/D3 treated with different concentrations of METH for 24 h. Using β-actin as an internal parameter, the control group was normalized to 1 and METH groups were compared with control group. The statistical significance is shown as * *p* < 0.05, ** *p* < 0.01, *** *p* < 0.001, **** *p* < 0.0001. All the data were derived from cell passages of at least three batches.

**Scheme 3:**
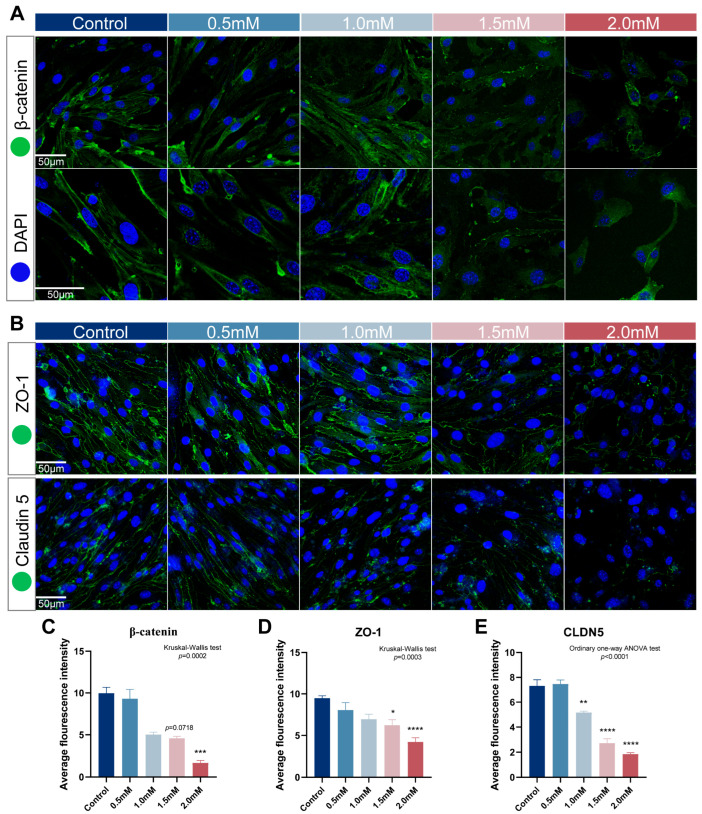
The increase of METH concentration causes decrease of β-catenin, ZO-1 and claudin5. (**A**) Immunofluorescence staining of β-catenin of bEnd.3 after METH concentration gradient treatment for 24 h. Images in upper row were captured at 400× magnification, while those in lower row were at 630× magnification. (**C**) Corresponding average fluorescence intensity statistics of β-catenin. (**B**) Immunofluorescence staining of ZO-1 and claudin5 of bEnd.3 after METH concentration gradient treatment for 24 h. (**D**,**E**) Corresponding average fluorescence intensity statistics of ZO-1 and claudin5. The statistical significance is shown as * *p* < 0.05, ** *p* < 0.01, *** *p* < 0.001, **** *p* < 0.0001.

Given our observation of BBB leakage and decreased β-catenin levels in chronic METH-exposed mice with cognitive impairment, we investigated the impact of METH on endothelial β-catenin and other connexins. We employed CCK8 assay to assess the effect of varying concentrations of METH on cell viability in two different brain microvascular endothelial cell lines: bEnd.3 (METH concentration gradients: 0, 0.5, 1.0, 1.5, 2.0, 2.5 mM) and hcmec/D3 (METH concentration gradients: 0, 0.5, 1.5, 2.5, 3.5, 4.5 mM). Our results demonstrate that, although these two endothelial cell lines exhibited different tolerances to METH concentrations, their respective viabilities decreased in a dose-dependent manner as the concentration of METH increased (Figure 8A). Subsequently, we employed the 70 kDa FITC-dextran permeability assay to assess the integrity of the monolayer endothelial barrier in bEnd.3 and hcmec/D3 cells. Remarkably, we observed a dose-dependent increase in endothelial barrier permeability upon exposure to escalating concentrations of METH in both cell types (Figure 8B). Furthermore, morphological analysis of bEnd.3 cells following 24 h treatment with a METH concentration gradient revealed pronounced cellular contraction and pseudopodia tapering, accompanied by distorted shapes with irregular widths. Additionally, there was a significant reduction in cell soma length and an inclination towards roundness. Swollen nuclei, cytoplasmic vesicles or pits, as well as widened intercellular areas were also evident (Figure 8C). Western blot analysis demonstrated alterations in endothelial connectin expression after METH concentration gradient treatment. Specifically, ZO-1, CDH5, β-catenin and claudin5 exhibited gradual decreases with increasing METH concentrations in bEnd.3 cells (Figure 8D). In hcmec/D3 cells, the expression levels of ZO-1, β-catenin, and claudin5 decreased with increasing METH concentration; however, CDH5 increased. Additionally, occludin showed a trend of slight decrease followed by an increase (Figure 8E). Immunocytochemistry staining further confirmed the reduction in β-catenin, ZO-1 and claudin5 proteins upon exposure to higher levels of METH (Scheme 3A–E).

**Figure 9:**
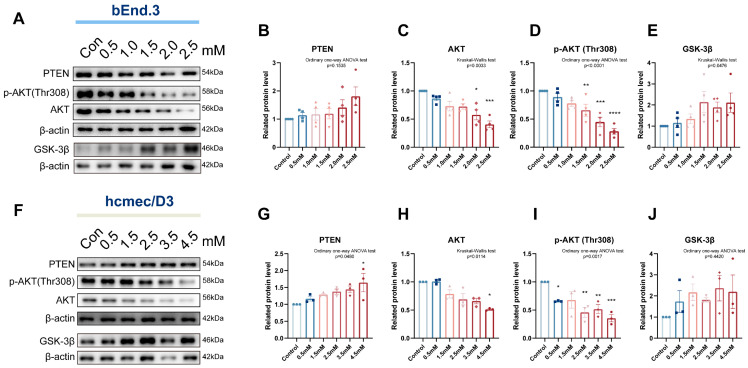
METH concentration gradient treatments affected PTEN/AKT/GSK-3β signal axis in endothelial cells. (**A**) Western blot of PTEN, p-AKT, AKT, GSK-3β in bEnd.3 treated by METH concentration gradient. (**B**–**E**) Relative protein level analyses of PTEN, p-AKT, AKT, GSK-3β in bEnd.3. (**F**) Western blot of PTEN, p-AKT, AKT, GSK-3β in hcmec/D3 treated by METH concentration gradient. (**G**–**J**) Relative protein level analyses of PTEN, p-AKT, AKT, GSK-3β in hcmec/D3. All the data were derived from cell passages of at least three batches. The statistical significance is shown as * *p* < 0.05, ** *p* < 0.01, *** *p* < 0.001, **** *p* < 0.0001.

In the cytoplasm, β-catenin can undergo phosphorylation by the degradation complex (involving GSK-3β) and subsequent degradation via the ubiquitin proteasome pathway. GSK-3β is a target of the AKT signaling pathway. Considering these findings, we also observed the impact of METH concentration gradients on the PTEN/AKT/GSK-3β signaling axis in endothelial cells. In bEnd.3 cells treated with METH concentration gradients (Figure 9A), an increase in METH led to a decrease in p-AKT and AKT levels (Figure 9C,D), while GSK-3β exhibited an upward trend (Figure 9E). The negative regulator of the AKT signaling axis, PTEN, also showed an increasing trend (Figure 9B). Similar changes were observed in hcmec/D3 cells following METH concentration gradient treatments (Figure 9F). As METH concentrations increased, both p-AKT and AKT levels decreased (Figure 9H,I), accompanied by an increasing trend in GSK-3β expression (Figure 9J), along with up-regulation of PTEN expression (Figure 9G).

### 2.5. METH Treatment Duration Induced the Decrease of β-Catenin and Its Transfer from Cytomembrane to Nucleus

**Figure 10:**
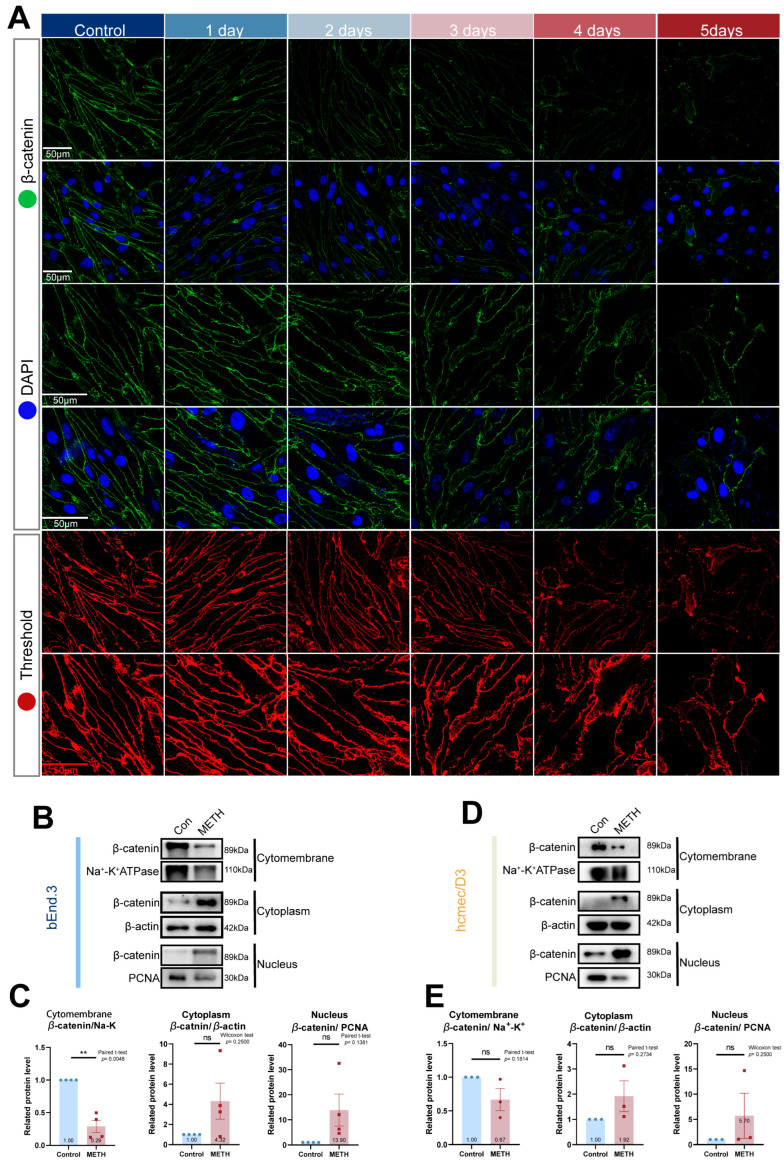
METH treatment duration induced the decrease of β-catenin and its transfer from cytomembrane to nucleus. (**A**) The immunofluorescence staining of β-catenin in bEnd.3 after continuous treatment with 1.0 mM METH for 0~5 days (400× and 630× magnification). The threshold images show decreased cytomembrane β-catenin and increased cytoplasm β-catenin. (**B**,**C**) Cell membrane, cytoplasmic and nuclear components of bEnd.3 were separated and β-catenin expression levels of each component were detected by Western blot, and corresponding statistics. (**D**,**E**) The same detection and statistics of hcmec/D3. All the data were derived from cell passages of at least three batches. The statistical significance is shown as ns *p* > 0.05, ** *p* < 0.01.

**Scheme 4:**
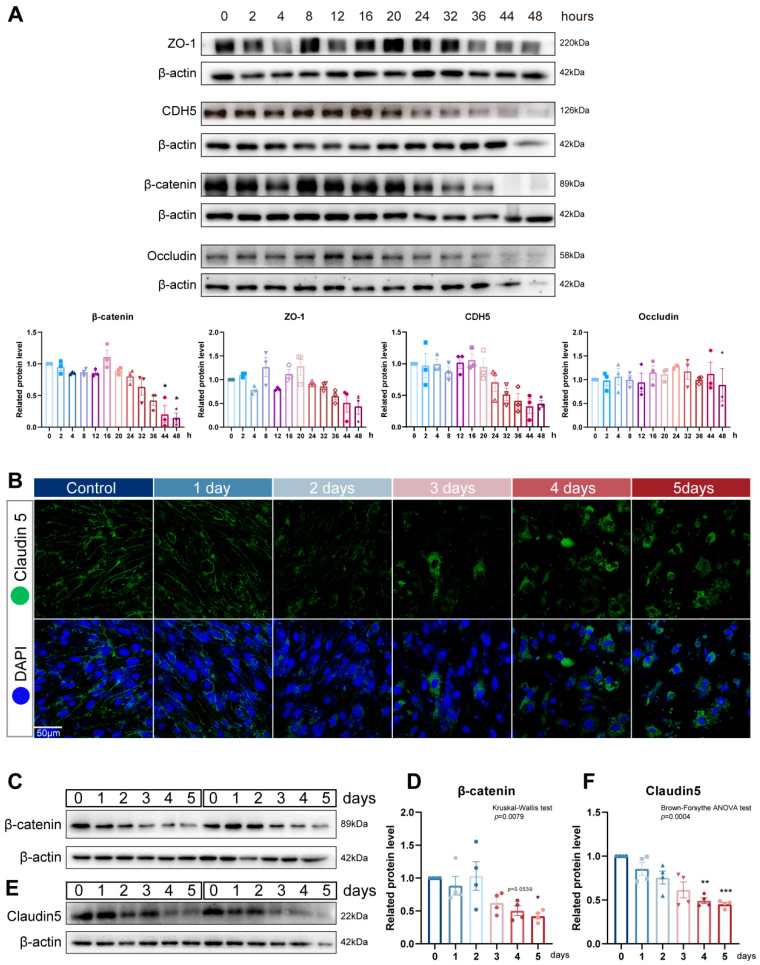
METH treatment duration induced the decrease of β-catenin and other junction proteins. (**A**) Western blot analysis of ZO-1, CDH5, β-catenin and occludin in bEnd.3 treated by 1.5 mM METH for a 0–48 h time gradient. (**B**) Immunofluorescence staining of β-catenin in bEnd.3 after continuous treatment with 1.0 mM METH for 0~5 days (400× magnification). (**C**,**D**) Western blot analysis of β-catenin in bEnd.3 after continuous treatment with 1.0 mM METH for 0~5 days. (**E**,**F**) Western blot analysis of claudin5 in bEnd.3 after continuous treatment with 1.0 mM METH for 0~5 days. All the data were derived from cell passages of at least three batches. The statistical significance is shown as * *p* < 0.05, ** *p* < 0.01, *** *p* < 0.001.

In addition to concentration, the duration of METH may also influence the expressions of junction proteins in endothelial cells. We selected a concentration of 1.5 mM METH and treated bEnd.3 cells for varying durations ranging from 0 to 48 h. Western blot analysis was employed for detection (Scheme 4A). The expression level of ZO-1 showed fluctuations between 0 to 16 h, followed by a gradual decrease from 20 to 48 h. β-catenin exhibited a slight decrease at 0–4 h, remained unchanged at 4–12 h, increased at 16 h and gradually decreased at 16–48 h. CDH5 displayed minimal fluctuations within 0–16 h and then gradually decreased in 16–48 h. Occludin demonstrated little change up until 8 h, with a slight decrease observed at 12 h; it increases slightly at 16–44 h (except 36 h), before decreasing again at 48 h. To mimic chronic administration conditions, bEnd.3 cells were continuously exposed to a low dose of METH (1.0 mM) over five days, and the expression levels of β-catenin and claudin5 were evaluated using immunofluorescence staining and Western blot analysis. Our findings demonstrate that prolonged METH treatment resulted in decreased levels of β-catenin protein in endothelial cells, accompanied by alterations in its subcellular localization (Figure 10A, Scheme 4C,D). Moreover, examination of fluorescent images depicting β-catenin distribution patterns on cell membranes under increasing durations of METH exposure demonstrated diminishing signals with discontinuity while scattered signals appeared more prominently within the cytoplasmic region (Figure 10A). Similar changes were observed in the expression pattern of claudin5 compared with β-catenin (Scheme 4B,E,F).

To confirm the reduction of β-catenin on the cytomembrane and changes in subcellular localization, we isolated the cell membrane, cytoplasm, and nucleus from bEnd.3 cells treated with 1.5 mM METH for 48 h, as well as hcmec/D3 cells treated with 2.5 mM METH for 48 h. Subsequently, we evaluated the expression levels of β-catenin in each cellular compartment. The results demonstrate a significant decrease in β-catenin expression on the cytomembrane component while an increase was observed in its expression within the cytoplasm and nucleus of bEnd.3 cells (Figure 10B,C). Similar observations were made in hcmec/D3 cells where β-catenin decreased on the cytomembrane but increased within the cytoplasm and nucleus (Figure 10D,E).

### 2.6. The Decrease of β-Catein Is Detrimental to Endothelial Cell Junction While Overexpress of β-Catein Can Reduce the Endothelial Injury Effect of METH

**Figure 11:**
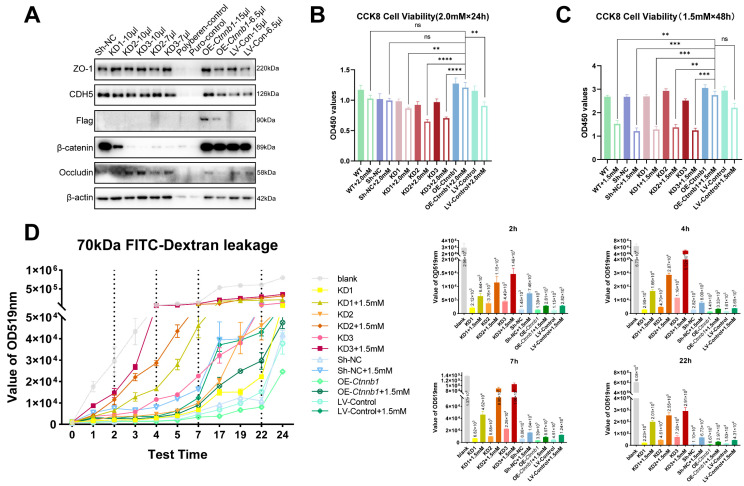
Effects of knockdown and overexpression of β-catenin on cell activity and permeability of bEnd.3. (**A**) The knockdown and overexpression of β-catenin were detected by Western blot. (**B**,**C**) CCK8 was used to detect the cell viability of knockdown and overexpressed cell lines and the viability under treatments of 2.0 mM METH treating for 24 h and 1.5 mM METH treating for 48 h. (**D**) The detection of 70 kDa using an FITC-dextran permeability test for monolayer endothelial barrier of different cell lines and for those under 1.5 mM METH treatment for 48 h. The statistical significance is shown as ns *p* > 0.05, ** *p* < 0.01, *** *p* < 0.001, **** *p* < 0.0001.

To investigate the impact of β-catenin on endothelial junctions, knockdown and overexpression cell lines were generated using bEnd.3 cells to evaluate their permeability under normal conditions and after METH treatment. Western blot analysis was employed to validate both knockdown and overexpression effects of β-catenin protein levels within these cell lines. The results demonstrate a graded reduction in β-catenin expression levels in the knockdown group (KD), with KD3 showing the lowest expression followed by KD2 and KD1, while successful overexpression of β-catenin with the flag tag was observed in the overexpression cells (OE). Notably, interference with β-catenin did not affect junction proteins such as ZO-1, CDH5, or occludin (Figure 11A).

Cell viability comparisons among different knockdown or overexpression cell lines were conducted using CCK8 assays before and after METH treatments. There was no significant change of cell viability between KD, OE and wild type (WT) cell lines before METH treatment. However, after treatment with 2.0 mM METH for 24 h, the cell viability of KD groups decreased significantly compared with the OE-*Ctnnb1* group which showed a slighter decrease (Figure 11B). After treating cells with 1.5 mM METH for 48 h, both KD and WT groups exhibited a significant decrease in cell viability while the decrease observed in the OE-*Ctnnb1* group was less pronounced than that seen in the KD and WT groups (Figure 11C). The cell morphology was observed using an inverted microscope. Untreated Ctnnb1 knockdown resulted in a decrease in the uniformity of cell arrangement, enlargement of intercellular spaces, rounding of cells, and contraction of pseudopodia.

An FITC-dextran permeability test was conducted to assess the monolayer endothelial permeability of different cell lines under PBS/1.5 mM METH treatment for 48 h. By analyzing the fluorescent signals that penetrated into the lower chamber at various time points, we observed that, compared with groups without METH, KD2 (bright orange) and KD3 (bright red) exhibited higher levels of ventricular FITC leakage, with KD3 showing a more pronounced increase. Following METH administration, although all groups showed increased permeability, the rise in FITC leakage was greater in the KD groups and followed the following trend: KD3 (dark red) > KD2 (dark orange) > KD1 (dark yellow), while the OE-*Ctnnb1* group displayed less leakage (dark green) (Figure 11D, where the results of 2, 4, 7 and 22 h are listed on the right).

**Figure 12:**
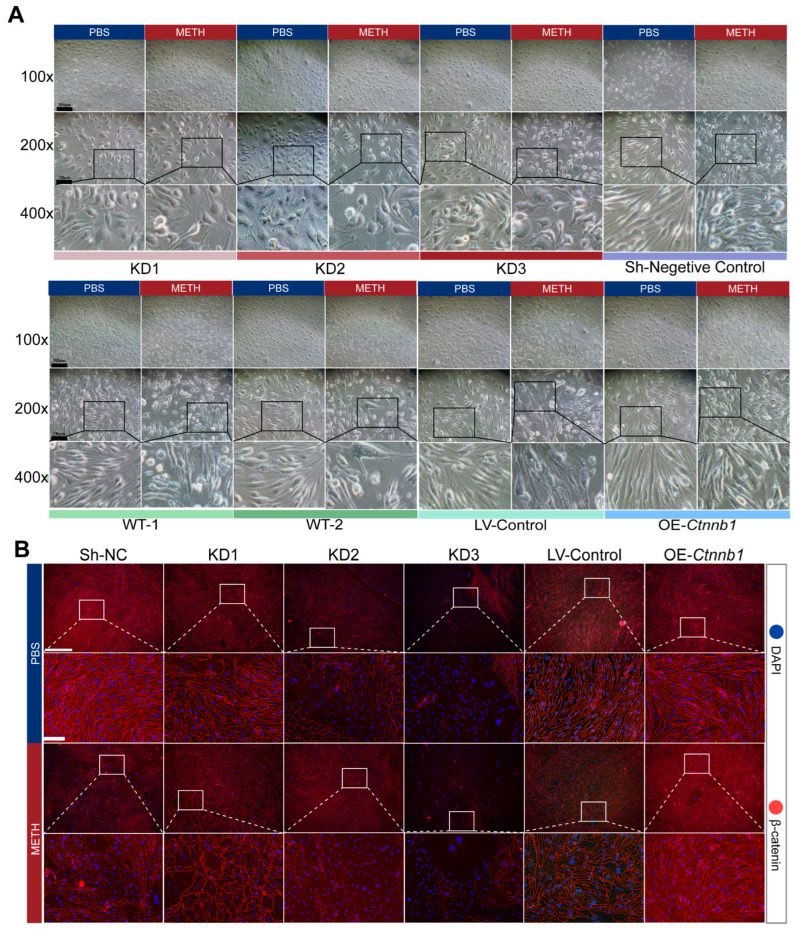
Effects of knockdown and overexpression of β-catenin on morphology of bEnd.3. (**A**) Morphological observation of different cell lines under 2.0 mM METH treatment for 24 h, scale bars: 500 μm for 100×, 250 μm for 200× and 400× images were enlarged twice from 200×. (**B**) Immunocytochemistry of β-catenin in different cell lines on the transwell after FITC-dextran permeability test. The upper images of each group are formed by scanning and stitching together at a magnification of 200×, while the lower images are displayed by magnifying three times within a rectangular selection area. Scale bars represent 200 μm for upper and 50 μm for lower, respectively.

Subsequently, after treatment with 2.0 mM METH for 24 h, endothelial cells in each group exhibited varying degrees of cell contraction, irregular shape, zigzagging of cell membrane edges, atrophy of pseudopodia, widening of perinuclear circumference and increased vesicles formation. Additionally, there was an increase in the number of apoptotic cells with enhanced refraction and widening intercellular spaces, along with other toxic changes observed. These changes were more pronounced in KD1, KD2 and KD3 groups, while being less significant in the OE-*Ctnnb1* group (Figure 12A).

After conducting the FITC permeability test, immunocytofluorescence analysis was performed to evaluate β-catenin expression in transwell monolayer endothelial barriers (Figure 12B). The fluorescence signal intensity indicated lower β-catenin expression levels in the KD groups without METH treatment, ranked as follows: KD3 < KD2 < KD1. Additionally, cellular morphology within these groups transitioned from slender deformation to diamond-shaped polygons and other irregular shapes, with disordered and irregular cell arrangements observed. Upon METH treatment, further reduction in β-catenin expression along with noticeable changes in cell morphology (from a narrow strip to a rhombus shape) and disorderly arrangement were evident within the KD groups; however, most cells remained elongated and tightly arranged within the OE-*Ctnnb1* group.

**Figure 13:**
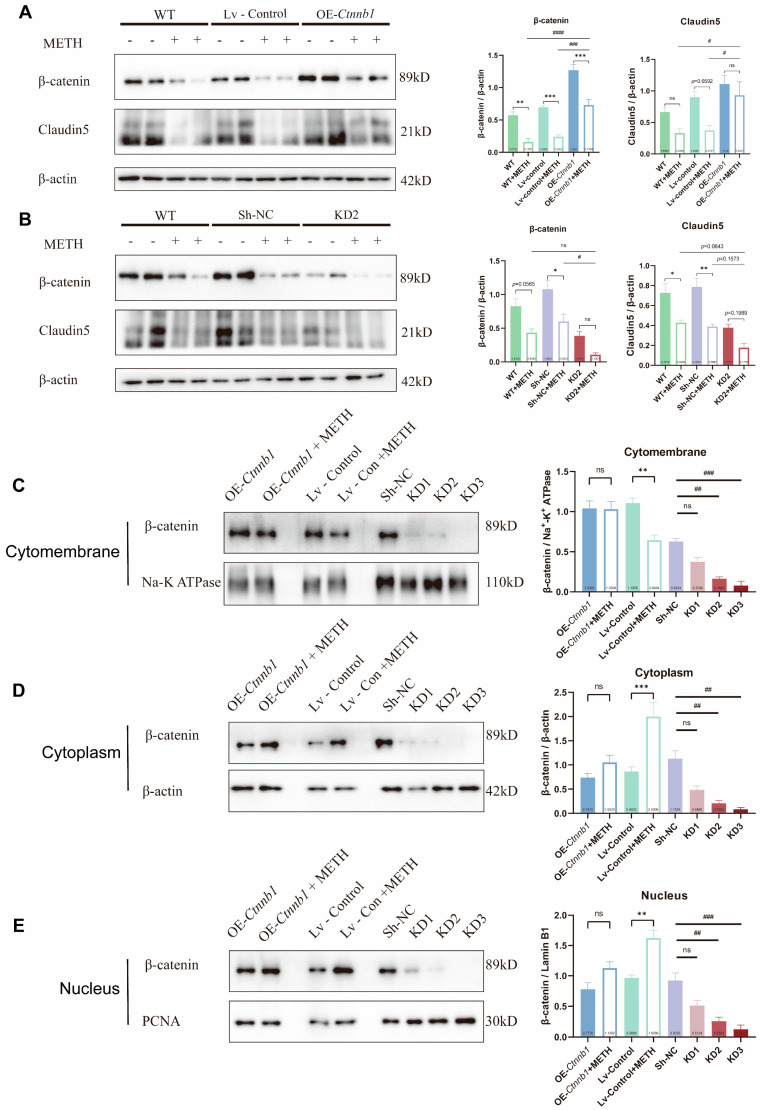
Effects of knockdown and overexpression of β-catenin on β-catenin and claudin5 protein levels, and β-catenin expression levels on cytomembrane, cytoplasm and nucleus. (**A**,**B**) Western blot analysis of β-catenin and claudin5 protein levels in cells subjected to knockdown, overexpression, and METH treatment. (**C**–**E**) Western blot analysis of β-catenin expression levels on cytomembrane, cytoplasm and nucleus in cells subjected to knockdown, overexpression, and METH treatment. In the statistical comparison of data corresponding to the two endpoints of the connection, symbols * and # are used to denote significant differences. The statistical significance is shown as: ns *p* > 0.05, * *p* < 0.05, ** *p* < 0.01, *** *p* < 0.001; # *p* < 0.05, ## *p* < 0.01, ### *p* < 0.001, #### *p* < 0.0001.

Furthermore, we treated overexpression and knockdown cell lines with 1.5 mM METH and found, through Western blot analysis, that β-catenin overexpression alleviates the METH-induced suppression of β-catenin and claudin5 expression in bEnd.3 (Figure 13A); however, in the knockdown group, METH treatment could lead to a decrease in the expression of both β-catenin and claudin5 (Figure 13B).

We also detected the changes in the expression levels of β-catenin in the membrane, cytoplasm and nuclear components of the knockdown and overexpression cell lines. Among the components of the cytomembrane, overexpression of β-catenin alleviated the reduction of β-catenin in the cell membrane caused by METH, while knockdown directly led to a decrease in the expression of β-catenin in cytomembrane (Figure 13C). Meanwhile, overexpression of β-catenin appears to exert an ameliorative effect on the accumulation of β-catenin in the cytoplasm and nucleus in response to METH treatment (Figure 13D,E).

## 3. Discussion

An increasing number of studies have focused on investigating the impact of METH on cognitive function [18,19,20]. Clinical evidence suggests that METH abusers exhibit significantly lower cognitive scores compared with non-abusers, with learning, executive function, memory, spatial cognition, and processing speed being particularly affected [21,22]. However, it is challenging to determine whether the observed cognitive dysfunction is preexisting or a consequence of behaviors associated with general METH use disorders in cross-sectional studies. Additionally, METH-induced cognitive impairments are influenced by various factors and may not always be significant [18]. Animal experimental evidence indicates that animals exposed to METH (especially those mimicking human abuse patterns) demonstrate cognitive impairment; however, results can vary depending on dosage, duration of exposure, and administration methods [23]. In our study, we employed a gradual incremental model for administering METH (with daily doses ranging from 1 to 10 mg/kg/day i.p. over a period of 28 days) and utilized behavioral tests with reduced stress levels to evaluate the cognitive function of mice. Our findings reveal that chronic exposure to METH in mice leads to deficits in working memory and spatial memory as well as decreased interest in exploration. Moreover, it affects overall cognitive processes and increases susceptibility to anxiety-like emotions. Extensive research consistently emphasizes the pivotal role of the hippocampus in the storage and retrieval of new memories, as well as spatial navigation [24], and the disruption of BBB integrity initiates in the hippocampus during the pathological progression associated with cognitive impairment [25]. In addition, we found there was a significant difference in the Y-maze test, so we chose hippocampus as the target brain region.

Extensive evidence suggests that injury to the BBB can lead to neuroinflammation and neural damage, as the BBB facilitates the transport of specific nutrients from peripheral circulation to the central nervous system (CNS), while preventing the entry of unnecessary or harmful substances [26]. In neurodegenerative diseases such as AD, an increase in Aβ and Tau tangles is a well-established pathological manifestation. Our findings demonstrate elevated levels of APP and phosphorylated tau in the hippocampi of mice chronically exposed to METH, indicating neuronal damage associated with cognitive impairment. Immunohistochemical staining for Aβ1–42 revealed amyloid accumulation in the hippocampus following chronic METH exposure, further suggesting impaired clearance of Aβ near blood vessels, potentially involving endothelial cells.

When the BBB is damaged, a significant influx of leukocytes, fibrinogen, immunoglobulins, and cytokines from peripheral blood occurs, leading to increased BBB permeability and subsequent neuroinflammation. This persistent neuroinflammation can also contribute to BBB disruption. The main inflammatory factors responsible for disrupting the tight junctions, particularly claudin5, are IL-1β, IL-6, and TNF-α. Additionally, IL-6 negatively regulates the Wnt signaling pathway for which β-catenin plays a crucial role [27]. In our study, we observed a significant increase in IL-6 and TNF-α levels in the hippocampus of mice exposed to METH for 2 weeks; whereas in mice exposed for 4 weeks, there was a significant increase in IL-1β and TNF-α levels. Considering that astrocytes regulate neuroinflammation and play an important role in BBB integrity maintenance, we found activated astrocytes through GFAP immunohistochemical staining in the hippocampus of chronically METH-exposed mice. Furthermore, ELISA results measuring p-Tau, NEFL, and GFAP levels in plasma indicated neural damage and neuroinflammation caused by METH exposure within the CNS. These findings provide potential peripheral markers for monitoring METH-induced neural damage.

MRI scanning and Evans blue leakage were used to prove increased BBB permeability in METH exposed mice. Different from some studies of METH, our model revealed that the increase in BBB permeability is similar to microleakage, with limited contrast media escaping, in turn meaning that blue staining was not visible throughout the entire brain. We believe that it is necessary to remove excess dye from blood vessels by perfusion and to observe the leakage under a microscope. Nevertheless, we still observe that Evans blue was internalized by neural cells under a microscope, which may also lead to its residual presence in the central nervous system. We investigated a range of junction-related proteins both in vitro and in vivo; however, not all protein trends were found to be correlated with reduced connectivity between endothelial cells. Therefore, we propose that further consideration and experiments are necessary regarding the heterogeneity of protein expression abundance across distinct cell types within tissues, the dynamic alterations of nexins in response to stimulation, and the diverse functionalities of nexins in BBB junction structures (e.g., molecular weight filtration). Among these factors, we observed a strong correlation between METH and the downregulation of β-catenin as well as its regulated claudin5. This is in line with our finding that, in endothelial cells with knocked-down and overexpressed β-catenin, there is a certain correlation between the expression levels of β-catenin and claudin5. Although Western blot analysis did not show a significant decrease in claudin5 levels in the hippocampi, co-staining and vascular tracing with Cd31 and claudin5 demonstrated reduced expression of claudin5 on endothelial cells in chronically METH-exposed mice. The discrepancy in claudin5 results may be attributed to its expression in other cell types within the hippocampus, such as mural cells (searching “mouse” and “claudin5” on the website CellMarker 2.0 [28]).

As we observed a significant downregulation of β-catenin, a protein associated with adherens junctions, in the hippocampi of mice exposed to METH for both 2 and 4 weeks, and which may be linked to BBB impairment, our hypothesis is that the reduction of β-catenin in endothelial cells under METH exposure leads to BBB dysfunction. Cerebrovascular endothelial cells are crucial components in the formation of BBB, accounting for over 85% of cerebrovascular length and playing an indispensable role as selective permeability barriers [29]. As an intracellular ligand of cadherin, cytomembrane-localized β-catenin plays a pivotal role in adherens junction formation, which is essential for tight junction assembly and triggers various intracellular changes such as cytoskeleton reorganization, cell polarity establishment, lipid composition modulation of plasma membrane, transport facilitation and tight junction protein assembly, etc. [30,31]. We discovered that both the concentration and duration of METH exposure can decrease endothelial β-catenin levels, leading to increased permeability of the endothelial barrier; however, overexpression of β-catenin can rescue this augmented leakage. These findings suggest that BBB impairment induced by METH affects not only tight junctions but also endothelial cell adherens junctions. In the cytoplasmic compartment, GSK3-Axin-APC degradation complex phosphorylates β-catenin followed by its degradation through the ubiquitin-proteasome pathway. Considering that AKT signaling pathway might influence the GSK3β activity that affects the β-catenin degradation process, we also observed decreased expression levels of AKT and p-AKT upon METH exposure.

As a Wnt signal transduction molecule, β-catenin accumulates in the nucleus in response to Wnt signaling to regulate gene expression by interacting with other transcription factors. Several tight junction proteins (e.g., claudin3, claudin5); glucose transporters (Glut-1); matrix metalloproteinases (MMPs), fibronectins (FN, slug, laminin); proteins associated with cell growth, proliferation, and differentiation (c-Myc, c-jun, CCND); as well as transcription factors (TCF1, PPAR) are transcriptional targets of the Wnt/β-catenin pathway in endothelial cells. Previous studies on METH-induced BBB impairment have reported that a reduction and redistribution of claudin5 and an increase in MMP9 contribute to METH-induced BBB dysfunction [7]. Our findings regarding the translocation of β-catenin from the cytomembrane to the nucleus suggest that β-catenin-related signaling pathways may serve as upstream mechanisms for these changes. Furthermore, it is worth noting that the Wnt/β-catenin signaling pathway also plays a crucial role in mediating epithelial–mesenchymal transition (EMT) [32], which refers to the transformation of weakened epithelial or endothelial connectivity into mesenchymal characteristics associated with growth. EMT is important for development and healing processes and could potentially explain the balance between endothelial impairment and repair or angiogenesis observed during chronic METH-induced endothelial injury, thus shedding light on whether METH-induced BBB impairment is sustained or temporary.

Activation of the Wnt/β-catenin pathway through various approaches, such as agonist stimulation [33], activation of Wnt proteins or receptors (Unc5B/Nertin, L6-F4-2) [34,35], gene interference, and astrocyte secretion [36], has demonstrated protective effects on the blood–brain barrier (BBB). Conversely, Aβ oligomers directly target endothelial cells to inhibit their Wnt/β-catenin signaling pathway, thereby disrupting BBB function [37]. Although we found that overexpression of β-catenin is beneficial for endothelial cells when resisting the decline in connectivity and cell viability caused by METH, similar to the findings of Kevin Boye et al. [26], there are still many issues worth discussing, such as the activity of overexpressed β-catenin, its binding efficiency with transcription factors, and its influence on multiple signaling pathways.

Limitations and future directions: 1. We observed a decrease in β-catenin levels in bEnd.3 cells in vitro, consistent with the reduced β-catenin protein expression in the hippocampal tissues of chronic METH-exposed mice. However, direct evidence confirming β-catenin reduction in hippocampal endothelial cells remains lacking. Future studies should employ flow cytometry and other methods to isolate and analyze endothelial cells, as well as utilize AAVs such as BR1 or BI30 to specifically modulate β-catenin expression in hippocampal endothelial cells. This approach will facilitate the evaluation of its effects on neuroinjury and cognitive function. 2. The AKT/GSK3β signaling pathway and the Wnt signaling pathway are key regulators of β-catenin expression and function in endothelial cells. However, our current research in this area remains limited. Interventions using inhibitors, activators, or mimetics of these pathways may enhance the repair of cerebral vascular endothelial injury. Additionally, the Wnt signaling interactions between astrocytes, neural stem cells, and endothelial cells in the hippocampus may represent critical targets for investigation.

## 4. Materials and Methods

### 4.1. Animal and Treatments

All protocols were approved by the Institutional Animal Care and Use Committee of Southern Medical University (Ethical number: L2022125) and were consistent with NIH Guidelines for the Care and Use of Laboratory Animals (8th Edition, U.S. National Research Council, 2011). Healthy male mice (7 weeks old) on C57BL/6 background were purchased from the Laboratory Animal Center of Southern Medical University (Guangzhou, China) and were housed in a central exhaust ventilation cage system (Houhuang Experimental Equipment Technology, Suzhou, China) with a 12/12 h light–dark cycle. The temperature of system was set at 20–22 °C and the humidity was set at 45–55%. Mice had access to water and food at will and were kept for up to 10 weeks before treatments or experiments in order to eliminate the effects of transportation stress and environmental changes.

**Figure 14:**
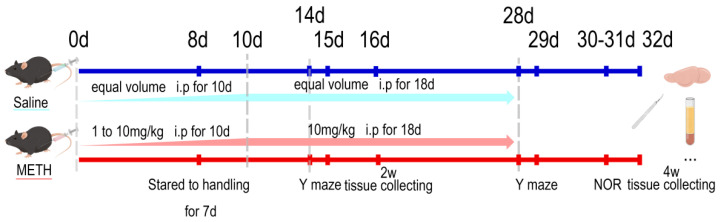
Establishment of chronic METH exposure model and behavioral test and sampling arrangement.

Building upon the prior drug administration protocols established in our laboratory [38,39,40,41], we implemented several enhancements and adopted a progressive dosing regimen. This approach was designed to effectively induce cognitive deficits while mitigating potential adverse reactions associated with medium and high doses of METH [42]. Mice were randomly divided into a saline group as control and a METH group as experiment. Mice of the METH group were treated intra-peritoneally with METH (National Institutes for the Control of Pharmaceutical and Biological Products, Beijing, China) dissolved in saline at an incremental dose from 1 up to 10 mg/kg (twice a day for 14 or 28 days as shown in Figure 14). Mice of the saline control group simultaneously underwent the same manner of injection but with saline. The last injection administered 12 h prior to the animal behavior test or sample collection will be skipped.

### 4.2. Animal Behavioral Tests

After mice were administered METH for 2 or 4 weeks, they underwent behavioral assessment.

Y maze test: Y maze tests were conducted at the end of 2 and 4 weeks of METH administration to assess working memory and spatial cognition in mice. This test was based on the initial method proposed by Lester, D. and Greenberg, L. A. [43], and was improved as described by Martina Riva et al. [44]. The Y-maze test box was composed of three exploration arms (length 40 cm, width 10 cm, height 15 cm) at 120° angles to each other. The arms were pasted with frosted cellophane. Mice were placed in a Y-maze from the end of a fixed arm and then allowed to freely explore the entire Y-maze for 8 min, while the camera was located directly above the maze to record the movement behavior of the mice. After each test, the area used by the animals would be cleaned thoroughly with alcohol. The total number of entries of the exploration arm (recognizing all four legs of the mouse entering the exploration arm as the entry standard), the number of alternations (the mouse entering all three of the arms of the Y-maze once in a row) and their proportion (the number of alternations/total entries) were used as statistical indicators. The number of entries on different exploration arms and the percentage of alternation were counted for statistics.

Novel object recognition (NOR) test: The NOR test was conducted at the end of 4 weeks of METH administration to investigate learning and cognitive memory in mice. This test was based on the initial method proposed by A. Ennaceur et al. [45], and was improved as described by Martina Riva et al. [44]. The test room was illuminated by four LED lights, each of which was the size of the test boxes (50 cm × 50 cm × 40 cm) so as to ensure that the light intensity in the box was about 20 lx. In the adaptation stage, before the start of the test, the mice were placed in the test room for 24 h to acclimate, and they were given 10 min to explore the object-free test box, and were then returned to the resting cage. During the training stage, two identical looking objects were placed symmetrically in the test chamber and the mice were then allowed to explore freely for 10 min to fully familiarize themselves with the object. After training, the mice were returned to their resting cages to rest for 30 min. Then, during the testing stage, mice were sent back to their original test chamber where the old familiar object and a novel object were placed in the same location, and they were allowed to explore freely for 10 min. Familiar objects and novel objects and their position in each group were uniformly balanced. A camera system was placed above the test box to record the behavior of the mice in the test box for statistics. For mice, the following behaviors were considered as effective exploration and counted as exploration time: biting, sniffing, licking, touching objects with the nose or forelimb, or proximity between the nose and the object (≤1 cm). If the mouse put its hind legs on the object, or stood on the object, or did not move at all, then the exploration time was not counted. After each test, the used area and objects would be cleaned thoroughly with alcohol. The preference index for new objects was calculated as the time spent exploring new objects/total time spent exploring two objects and the discrimination index was calculated as the (time spent exploring new objects − time spent exploring old familiar objects)/total time spent exploring two objects. The frequency that each object was explored was counted, with average exploration duration = total exploration duration/frequency. Behavior was scored on video by two observers blinded to the mice’s treatment.

### 4.3. Magnetic Resonance Imaging (MRI) Scans

Referring to the methods of Caroline Menard et al. [46], following 4 weeks METH administration, blood–brain barrier (BBB) permeability was evaluated using a PharmaScan70/16 US (Bruker Biospin MRI GmbH, Rheinstetten, Germany) scanner system located at the SMU Central Laboratory. Mice were imaged under isoflurane anesthesia (3–4% in air for induction and 1.5% maintenance). Baseline T1 weighted images (T1-WIs) were acquired using a T1map_RARE sequence (about 20 slices, thickness 0.7 mm, in-plane resolution (256 mm × 256 mm)). An amount of 0.3 mmol/kg gadopentetate dimeglumine (Gd-DTPA) was intravenously injected into mice after finishing baseline scanning. Then, 25 min after injection, mice were anesthetized and rescanned using the same T1 protocol. Images were analyzed by Paravision (version 6.0.1, Bruker Biospin MRI GmbH, Rheinstetten, Germany). Regions of interest (ROIs) of encephalic regions were manually defined on the images and T1-WI mean image intensities were extracted by Paravision (Scheme 2). Signal enhancement was defined as (Mean_25min after injection_ − Mean_Basline_)/Mean_Basline_.

### 4.4. Immunohistochemistry Staining (IHC)

After being placed in deep anesthesia by intravenous injection of 40 mg/kg of pentobarbital sodium, mice were quickly sacrificed and perfused with PBS and 4% paraformaldehyde solution (4% PFA) for 5 min. Their brains were quickly and completely dissected on ice and placed in sufficient amounts of 4% PFA to fix for 24 h. The mice brains were dehydrated by gradient and immersed in paraffin and then embedded in paraffin blocks. The paraffin-embedded hippocampal tissues were sectioned into 5 μm slices on the coronal plane, and were then dewaxed, rehydrated, antigen retrieved and blocked with goat serum or 1% BSA. The slices were immunostained by β-amyloid 1-42 (ab201060, 1:1000, Abcam, Shanghai, China) and GFAP (ab68428, 1:250, Abcam, Shanghai, China). Then, these slices were incubated with biotinized secondary antibody and HRP labeled streptavidin of an SP Rabbit and Mouse HRP Kit (CW2069, CWBIO, Taizhou, China) to visualize protein signals. After being dehydrated and sealed, slices were viewed by a Zeiss microscope (Zeiss, Jena, Germany), and images were acquired by the corresponding Zeiss software (Version: 3.4 (blue edition), Zeiss, Jena, Germany). The end-point voxels analysis was undertaken per the article of Kimberly Young [47]. Image analysis was performed by Fiji-ImageJ (Version: 1.54f, Wayne Rasband and contributors, National Institutes of Health, Bethesda, MD, USA) [48]—AnalyzeSkeleton (Version: 3.4.2, an open-source software initially developed by Ignacio Arganda-Carreras.).

### 4.5. Immunofluorescence (IF) and Quantification of Claudin5 Levels

After being placed under deep anesthesia, mice were sacrificed by hemorrhagic shock and were then transcardially perfused with PBS and 4% PFA for 5 min each. Brain samples were dissected integrally and preserved in sufficient amounts of 4% PFA for 24 h and were then fully dehydrated in a 30% sucrose solution until they sank to the bottom. After being embedded in an optimal cutting temperature compound (OCT), the brain samples were sectioned into 40 μm slices in the coronal direction according to The Mouse Brain in Stereotaxic Coordinates (second edition, George Paxinos & Keith B. J. Franklin, 2001) [49]. The slices were preserved at −80 °C or used for staining. The slices went through unfolding, antigen retrieving, and permeabilizing by 0.3% Triton-X solution and blocking by 5% goat serum and were then incubated with the primary antibody CD31 (ab282746, 1:250, Abcam, Shanghai, China) and dissolved in 1% BSA with 0.05% Triton-X at 4 °C for 48 h to localize blood vessels. After five washes in PBS for 8 min, the slices were incubated away from light with fluorescent secondary antibody anti-rabbit-555 (A-21428, 1:500, Invitrogen, Shanghai, China). After five washes in PBS for 8 min, the slices were double immunostained with claudin5 (35–2500, 1:200, Invitrogen, Shanghai, China) similar to the first staining but lucifugally. After the same number of washes, the slices were incubated away from light with anti-mouse-488 (A-11001, 1:500, Invitrogen, Shanghai, China), and then washed again five times and stained with 4,6-diamidino-2-phenylindole (DAPI) to visualize nuclei. After staining, the slices were mounted and cover slipped with antifade mounting medium (P0131, Beyotime, Shanghai, China). Images of the hippocampus were acquired by laser confocal microscope FV3000 (Olympus, Tokyo, Japan) using a 10× and 40× lens with a resolution of 1024 × 1024. The z-stack images were optimized by the OLYMPUS FV31S-SW software (Version: 2.4.1.198 (Powered by H-PF Version 2.7.2.435), Olympus Corporation, Tokyo, Japan) according to the thicknesses and fluorescence signals of slices. The average intensity of flattened z-stack to x-y plane and its colocating parameter with the region or interested (ROI) defined using CD31 staining were compared using Fiji-ImageJ (V: 1.54f) colocalization analysis.

### 4.6. Enzyme-Linked Immunosorbent Assay (ELISA)

Phosphorylated Tau (p-Tau), glial fibrillary acidic protein (GFAP) and neurofilament light polypeptide (Nfl/NEFL) in mice serum were measured by ELISA kits (p-Tau: E-EL-M1289; GFAP: E-EL-M0554; NEFL: E-EL-M0817; Elabscience, Wuhan, China) according to the manufacturer’s instructions. Briefly, the sample and the double dilution standard were incubated in the enzyme-labeled plate and solidified. They were then combined with the antibody and detection reagent. Finally, a microplate reader (Synergy H1, BioTek, Santa Clara, CA, USA; software Version: 3.08.01) was used to detect optical density (OD) values at 450 nm to calculate the corresponding protein levels. “Origin” software (Version: 2022 (9.9), OriginLab Corporation, Northampton, MA, USA) was used to fit standard curves and calculate concentrations.

### 4.7. Fluorescence Detection of Evans Blue to Evaluate BBB Leakage in Hippocampus

Under isoflurane anesthesia, a 2% Evans blue solution (Sigma-Aldrich, St. Louis, MO, USA) in saline was intravenously administered to mice at a dose of 6 µL/g body weight, followed by a 16 h in vivo circulation period. For sampling, deeply anesthetized mice were first perfused with PBS for 5 min and subsequently with 4% PFA for 2 min. Brain samples were quickly removed, divided into 3 parts and preserved in 15 mL freshly prepared 4% PFA + 0.05% glutaraldehyde at 4 °C. After 36 h for fixation, the brains were fully dehydrated in a 30% sucrose solution until they sank to the bottom. The brains were sectioned as mentioned above. After being immersed and spread out, brain slices were laid flat on a slide and soaked with antifade mounting medium containing DAPI. After sealing, the slices were observed by laser confocal microscope FV3000 (Olympus, Tokyo, Japan) with lasers of 405 nm (DAPI) and 633 nm (or a longer wavelength, but within the emission wavelength range of Evans blue). The sectional scanning of the hippocampus area in mice is performed by a laser scanning microscope (BZ-X800LE, Keyence, Osaka, Japan) with the same laser. Fluorescent signals in tissues that were not flushed away by perfusion were regarded as leaking or as remaining Evans blue in brain parenchyma. The average intensity was measured by Fiji-ImageJ (Version: 1.54f).

### 4.8. Cell Culture and Treatment

Mouse brain microvascular endothelial cell line bEnd.3 (National Collection of Authenticated Cell Cultures of China, TCM40) was cultured in DMEM medium (Servicebio, Wuhan, China) containing 10% fetal bovine serum (ExCellBio, Suzhou, China, FSP500) and 1% penicillin/streptomycin (Gibco, Grand Island, NY, USA). Immortalized human brain microvascular endothelial cell line hcmec/D3 (presented by the Lyu Tianming Research Group of the Third Affiliated Hospital of Southern Medical University, Millipore, Burlington, MA, USA, SCC066) was cultured in DMEM/F12 (Gibco, San Francisco, CA, USA) medium containing 10% fetal bovine serum and 1% penicillin/streptomycin. Cells were kept in cell incubators (ThermoFisher, Shanghai, China) set at 37 °C and 5% CO_2_. The medium would be changed every other day.

Monolayer endothelial barrier was considered to be formed 1–2 d after endothelial cells were overgrown in the culture dish (endothelial cells were usually planted in a 6-well plate or a 6-well transwell at 1 × 10^6^ cell/insert) and their degree of convergence reached 100%. It usually took 6–7 d for bEnd.3 [50,51] and 3–4 d for hcmec/D3 [52]. Then, the monolayer endothelial barrier was used for METH treatments and following CCK8 (Gibco, USA), microscopic examination, FITC-dextran (Yeasen Biotechnology, Shanghai, China) permeability test, immunocytochemistry and Western blotting.

### 4.9. Assessment of Cell Viability

The evaluation of cell viability was conducted by the Cell Counting Kit-8 (CCK-8, Beyotime, Shanghai, China). Cells in the logarithmic phase of growth were dissociated, resuspended and adjusted to a density of 1 × 10^4^ per well and then planted uniformly in 96-well plates. After being cultured for 48 h, the cells were treated with METH of different concentrations and duration. After treatments, cells in each well received 10 μL CCK-8 solution and were incubated for 1 h before the assessment of OD values at 450 nm using a microplate reader (Synergy H1, BioTek, Santa Clara, CA, USA; software version: 3.08.01).

### 4.10. 70 kDa FITC (Fluorescein Isothiocyanate)-Dextran Permeability Test In Vitro

Monolayer endothelial barriers of bEnd.3 and hcmec/D3 were built on the upper chamber of a 6-well transwell. After METH treatments, 0.5 mg/mL 70 kD FITC-dextran (Yeasen Biotechnology, Shanghai, China) was added to upper chamber medium and blended gently and thoroughly, and then adequate culture mediums of the upper and lower chamber were replaced with fresh mediums. The culture systems were incubated in a cell incubator at 37 °C. After incubation for a specific amount of time, 50 μL culture medium samples from the upper and lower chamber were extracted to ELISA plate and the OD values at 490 nm (excitation) and 519 nm (emission) were measured by the microplate reader. The amounts of FITC penetration were calculated by reading and standard curve.

### 4.11. Western Blot Analysis

Total proteins of the hippocampus or cells were extracted using the RIPA containing 1% PMSF and 2% phosphatase inhibitors and mixed with a loading buffer (the above reagents were obtained from Beyotime Biotechnology, Shanghai, China), and then boiled to denature the protein. The proteins were separated using electrophoresis with 8–12% sodium dodecyl sulfate-polyacrylamide gel and transferred onto PVDF membranes (Millipore, Burlington, MA, USA). After being blocked for 2 h at room temperature, the membranes were incubated with the following primary antibodies dissolved in 5% BSA at 4 °C overnight: ZO-1 (21773-1-AP, 1:1000, Proteintech, Wuhan, China), VE-cadherin (CDH5, ab205336, 1:1000, Abcam, China), β-catenin (ab32572, 1:4000, Abcam, China), occludin (R381549, 1:1000, ZEN-BIOSCIENCE, Chengdu, China), β-actin (200068-6D7, 1:10000, ZEN-BIOSCIENCE, Chengdu, China), claudin5 (35–2500, 1:1000, Invitrogen, China; A10207, 1:1000, ABclonal, Wuhan, China), APP (A11019, 1:1000, ABclonal, Wuhan, China), Tau (sc-32274, 1:1000, Santa Cruz, Shanghai, China), p-Tau (Ser396) (sc-24597, 1:1000, Shanghai, China), p-Tau (T181) (ab254409, 1:1000, Abcam, China), IL-6 (A0286, 1:1000, ABclonal, China), IL-1β (A16288, 1:1000, ABclonal, China), TNF-α (26405-1-AP, 1:1000, Proteintech, Wuhan, China), BDNF (ab108319, 1:1000, Abcam, China), PTEN (9188, 1:1000, CST), AKT (4691, 1:1000, CST), p-AKT (Thr308) (13038, 1:1000, CST), and GSK-3β (sc-377213, 1:1000, Santa Cruz, Shanghai, China). After being washed and incubated with the secondary antibodies (115-035-003, 111-035-003, 1:10,000, Jackson ImmunoResearch, West Grove, PA, USA), the immunoblot bands were visualized by an ECL kit (Focus, Shanghai, China) and acquired on Tanon 5200 system (Tanon, Shanghai, China).

### 4.12. Knockdown and Overexpression of Ctnnb1

Gene targets were designed using the web-based tools of Thermo Fisher Scientific Inc., Shanghai, China [53].
**Primer Name****Primer Sequence (5′-3′)****Primer Length**shNC-oligo-FCCGGGCCACAACGTCTATATCATGGCTCGAGCCATGATATAGACGTTGTGGCTTTTTT58 bpshNC-oligo-RAATTCAAAAAAGCCACAACGTCTATATCATGGCTCGAGCCATGATATAGACGTTGTGG58 bp*Ctnnb1*KD1- TRCN0000012690-FCCGGGCTGATATTGACGGGCAGTATCTCGAGATACTGCCCGTCAATATCAGCTTTTTG58 bp*Ctnnb1*KD1- TRCN0000012690-RAATTCAAAAAGCTGATATTGACGGGCAGTATCTCGAGATACTGCCCGTCAATATCAGC58 bp*Ctnnb1*KD2- TRCN0000012692-FCCGGCCCAAGCCTTAGTAAACATAACTCGAGTTATGTTTACTAAGGCTTGGGTTTTTG58 bp*Ctnnb1*KD2- TRCN0000012692-RAATTCAAAAACCCAAGCCTTAGTAAACATAACTCGAGTTATGTTTACTAAGGCTTGGG58 bp*Ctnnb1*KD3- TRCN0000321075-FCCGGCGTGAAATTCTTGGCTATTACCTCGAGGTAATAGCCAAGAATTTCACGTTTTTG58 bp*Ctnnb1*KD3- TRCN0000321075-RAATTCAAAAACGTGAAATTCTTGGCTATTACCTCGAGGTAATAGCCAAGAATTTCACG58 bp

The target fragment was synthesized and then introduced into *Escherichia coli*. After purifying the monoclonal bacterial colony by PCR product identification, the colony was amplified and the plasmid was extracted. Transfection and virus packaging were performed by 293T cells, and then the virus were extracted and purified.

The overexpression procedure was roughly as follows: after amplifying the *Ctnnb1* gene, it was connected to the enzyme-cut vector and introduced into *E. coli*, as described above, for bacterial strain identification, plasmid extraction, and lentivirus packaging.

Prior to transfection, bEnd.3 cells were plated in 6-well plates and allowed to reach 70–80% confluency. Plasmid DNA was introduced using Lipofectamine 3000 transfection reagent according to the manufacturer’s protocol (Thermo Fisher Scientific). Following 24 h of transfection, the medium was refreshed with complete DMEM. Antibiotic selection with puromycin (2 μg/mL) was initiated 48 h post-transfection and maintained for three consecutive cycles to ensure clonal purity.

### 4.13. Data Analysis

All data are presented as mean ± SEM. Statistical tests select parametric tests or non-parametric tests according to normality tests and homogeneity tests of variance (when the data met the normal distribution and homoscedasticity, t-test or ANOVA test were used, and when the variances were not equal, methods of correction by test were used. When the data did not meet the normal distribution, the Mann–Whitney test or other non-parametric statistical methods were used), and multiple comparisons used the corrected post-test method. The *p* value < 0.05 was considered statistically significant and all statistical tests were two-tailed. Statistical tests were performed by the Prism software (v8.0.2, GraphPad Software Inc., San Diego, CA, USA).

## 5. Conclusions

**Figure 15:**
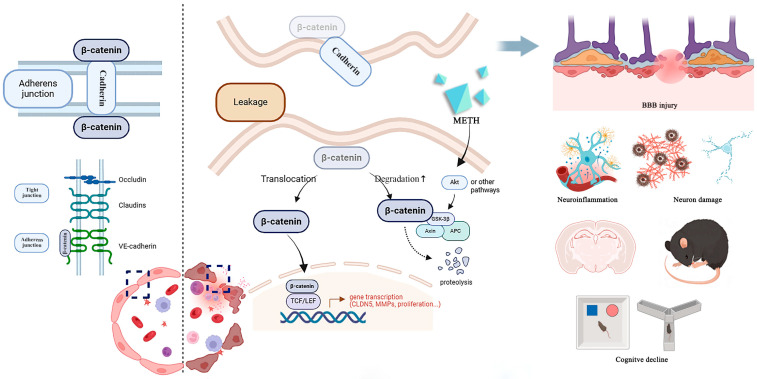
Concept drawing: METH diminishes endothelial β-catenin expression and induces its relocalization, compromising the integrity of the blood–brain barrier and eliciting cognitive impairment in chronically exposed mice. (The illustration is created with BioRender.com.)

In conclusion, our study provides compelling evidence that METH induces endothelial permeability and morphological alterations, compromising BBB integrity and leading to persistent neuroinflammation and neuronal damage in the hippocampus of mice with cognitive impairment from chronic METH exposure. The reduction in β-catenin levels in endothelial cells is closely associated with the METH-induced alteration of the AKT/GSK3β signaling axis. The translocation of β-catenin from the cell membrane to the nucleus likely plays a role in the transcriptional regulation of target genes, including claudin5. Overexpression of β-catenin mitigates the METH-induced decline in endothelial cell viability and connectivity, and aids in restoring claudin5 expression levels. As a result, the reduction in endothelial β-catenin levels induced by METH suggests potential therapeutic targets for addressing BBB impairment and cognitive deficits associated with chronic METH abuse (Figure 15).

## Data Availability

The data and materials of the study are available upon request from the corresponding author.
